# Supplementary material for: Upregulated Proteasome Subunits in COVID-19 Patients: A Link with Hypoxemia, Lymphopenia and Inflammation
Source: Biomolecules. 2022 Mar 13;12(3):442. doi: 10.3390/biom12030442 (PMC8946050; doi:10.3390/biom12030442)
Supplement: Supplementary file 1 [file biomolecules-12-00442-s001.zip › biomolecules-1638134-supplementary.pdf]

# Supplemental Information

# Upregulated Proteasome Subunits in COVID-19 Patients: A Link with Hypoxemia, Lymphopenia and Inflammation

**Enrique Alfaro [1,†], Elena Díaz-García [1,2,†], Sara García-Tovar [1], Ester Zamarrón [1,2], Alberto Mangas [1], Raúl Galera [1,2], Eduardo López-Collazo [3], Francisco García-Rio [1,2,4,*] and Carolina Cubillos-Zapata [1,2,*]**

[1] Respiratory Diseases Group, Respiratory Service, La Paz University Hospital, IdiPAZ, 28029 Madrid, Spain; quiquealfaro23@gmail.com (E.A.); elena.diaz.garcia.1994@gmail.com (E.D.-G.); sarugarto@gmail.com (S.G.-T.); ester.zamarron@gmail.com (E.Z.); mangasmoro@gmail.com (A.M.); raulgalera@hotmail.es (R.G.)

[2] Biomedical Research Networking Center on Respiratory Diseases (CIBERES), 28029 Madrid, Spain

[3] Innate Immune Response Group, IdiPAZ, 28029 Madrid, Spain; elcollazo@hotmail.com

[4] Faculty of Medicine, Autonomous University of Madrid, 28029 Madrid, Spain

* Correspondence: fgr01m@gmail.com (F.G.-R.); cubilloszapata@gmail.com (C.C.-Z.);
Tel.: +34-639911718 (F.G.-R.); +34-600877179 (C.C.-Z.)

† These authors contributed equally to this work.

**Table S1:** Primers used for qPCR

| | | |
|---|---|---|
| PSMA4 | Forward primer | CTTGTGAGCAGTTGGTTACAGCG |
| | Reverse primer | AGCCATAGTGCTTATCCCAGCC |
| PSMA 5 | Forward primer | TGCCATGTCTCGTCCCTTTG |
| | Reverse primer | TTTGTTGCATTCAGCTTCTCCT |
| PSMB1 | Forward primer | TGCAATGCTGTCTACAATCC |
| | Reverse primer | TCTCTGGTAAGACCCTACTG |
| PSMB2 | Forward primer | GACACTGTACAGTTTGCAGA |
| | Reverse primer | GGAGGTTCACATGATATGGG |
| PSMB3 | Forward primer | GAGCATGGTGGCCAACCTCT |
| | Reverse primer | GCAGGTGCCACTGACCACAA |
| PSMB4 | Forward primer | CGACTACGCTGATTTCCAG |
| | Reverse primer | GGTGTTCCACAAAGGGTTCA |
| PSMB5 | Forward primer | TGTAGCAGCTGCCTCCAAAC |
| | Reverse primer | CCCTGAAATCCGGTTCCCTT |
| PSMB6 | Forward primer | AGACTGGGAAAGCCGAGAAG |
| | Reverse primer | GCAGAAAATGCGGTCGTGAA |
| PSMB7 | Forward primer | AAACTGGCACGACCATCGCT |
| | Reverse primer | CAGCTGTCCCAGCACCACAA |
| PSMB8 | Forward primer | ACCCCGCGTGACACTACT |
| | Reverse primer | GGGACTGGAAGAATTCTGTGG |
| PSMB9 | Forward primer | CGTTGTGATGGGTTCTGATTCC |
| | Reverse primer | GACAGCTTGTCAAACACTCGGTT |
| PSMB10 | Forward primer | GGTTCCAGCCGAACATGA |
| | Reverse primer | GCCCAGGTCACCCAAGAT |
| PSMD11 | Forward primer | TAACTTCTGCTCGAACCACA |
| | Reverse primer | GTCGATGGAGTCATAACCCT |
| PSMD14 | Forward primer | GTGGATATCAACACTCAGCA |
| | Reverse primer | TGGTTCATGTCCTAAGACCA |
| PSME1 | Forward primer | GCGCTTGAAGCCTGAGATCA |
| | Reverse primer | CCTTCTCCTGGACAGCCACT |
| PSME2 | Forward primer | CTTTTCCAGGAGGCTGAGGAAT |
| | Reverse primer | GGAGGGAAGTCAAGTCAGCC |
| PSME3 | Forward primer | CCAGACCTAAGCTGCCTTCT |
| | Reverse primer | GATAGCAGCCTCTACTGGCA |
| HIF-1α | Forward primer | TTCCAGTTACGTTCCTTCGATCA |
| | Reverse primer | TTTGAGGACTTGCGCTTTCA |
| NF-κB | Forward primer | GGTGCGGCTCATGTTTACAG |
| | Reverse primer | GATGGCGTCTGATACCACGG |
| NLRP3 | Forward primer | TGCCCGTCTGGGTGAGA |
| | Reverse primer | CCGGTGCTCCTTGATGAGA |
| ASC | Forward primer | AACCCAAGCAAGATGCGGAAG |
| | Reverse primer | TTAGGGCCTGGAGGAGCAAG |
| CAS-1 | Forward primer | GGAAACAAAAGTCGGCAGAG |
| | Reverse primer | ACGCTGTACCCCAGATTTTG |
| GSDMD | Forward primer | GTGTGTCAACCTGTCTATCAAGG |
| | Reverse primer | CATGGCATCGTAGAAGTGGAAG |
| STAT3 | Forward primer | CAGCAGCTTGACACACGGTA |
| | Reverse primer | AAACACCAAAGTGGCATGTGA |
| c-FOS | Forward primer | GTGGGAATGAAGTTGGCACT |
| | Reverse primer | CTACCACTCACCCGCAGACT |
| c-JUN | Forward primer | GTCCTTCTTCTCTTGCGTGG |
| | Reverse primer | GGAGACAAGTGGCAGAGTCC |
| 18S | Forward primer | CGGCGACGACCCATTCGAAC |
| | Reverse primer | GAATCGAACCCTGATTCCCCGTC |

**Table S2.** Clinical parameters of COVID-19 patients grouped by proteasome subunits expression.

| | Lymphocytes (x10$^3$cells/mm$^3$) | | | PaO2/FiO2 ratio | | | C-reactive protein (mg/L) | | | Ferritin (mg/mL) | | |
|---|---|---|---|---|---|---|---|---|---|---|---|---|
| Gene | Low | High | P | Low | High | P | Low | High | P | Low | High | P |
| PSMB3 | 0.885±0.345 | 0.889±0.430 | 0.987 | 309.0±91.03 | 232.0±77.67 | 0.127 | 53.08±38.75 | 38.74±27.97 | 0.462 | 699.3±691.8 | 882±689.3 | 0.633 |
| PSMB5 | 0.997±0.357 | 0.742±0.339 | 0.213 | 290.6±98.11 | 257.1±88.33 | 0.503 | 47.99±32.83 | 43.08±32.03 | 0.775 | 867.4±620.8 | 699.0±713.6 | 0.633 |
| PSMB6 | 0.873±0.317 | 0.976±0.471 | 0.623 | 302.0±85.14 | 244.4±88.27 | 0.209 | 52.13±35.46 | 38.77±26.52 | 0.423 | 804.9±690.5 | 707.1±639.2 | 0.777 |
| PSMB7 | 0.873±0.317 | 0.976±0.471 | 0.623 | 302.0±85.14 | 244.4±88.27 | 0.209 | 52.13±35.46 | 38.77±26.52 | 0.423 | 804.9±690.5 | 707.1±639.2 | 0.777 |
| PSMD11 | 0.884±0.295 | 0.979±0.509 | 0.675 | 295.8±80.78 | 243.5±94.32 | 0.254 | 57.05±35.66 | 32.18±18.89 | 0.110 | 758.5±652.6 | 741.3±674.5 | 0.959 |
| PSMD14 | 0.884±0.295 | 0.921±0.498 | 0.858 | 295.8±80.78 | 240.3±88.77 | 0.197 | 57.05±35.66 | 42.19±34.85 | 0.400 | 758.5±652.6 | 667.0±669.1 | 0.780 |

Clinical parameters (Lymphocyte count, PAFI ratio, C-reactive protein concentration and ferritin concentration) comparison in COVID-19 patients grouped by high or low expression of proteasome subunits (PSMB3/5/6/7 and PSMD11/14). Data are mean±standard deviation. P-values are shown. Diferences were considered significant $p<0.05$.

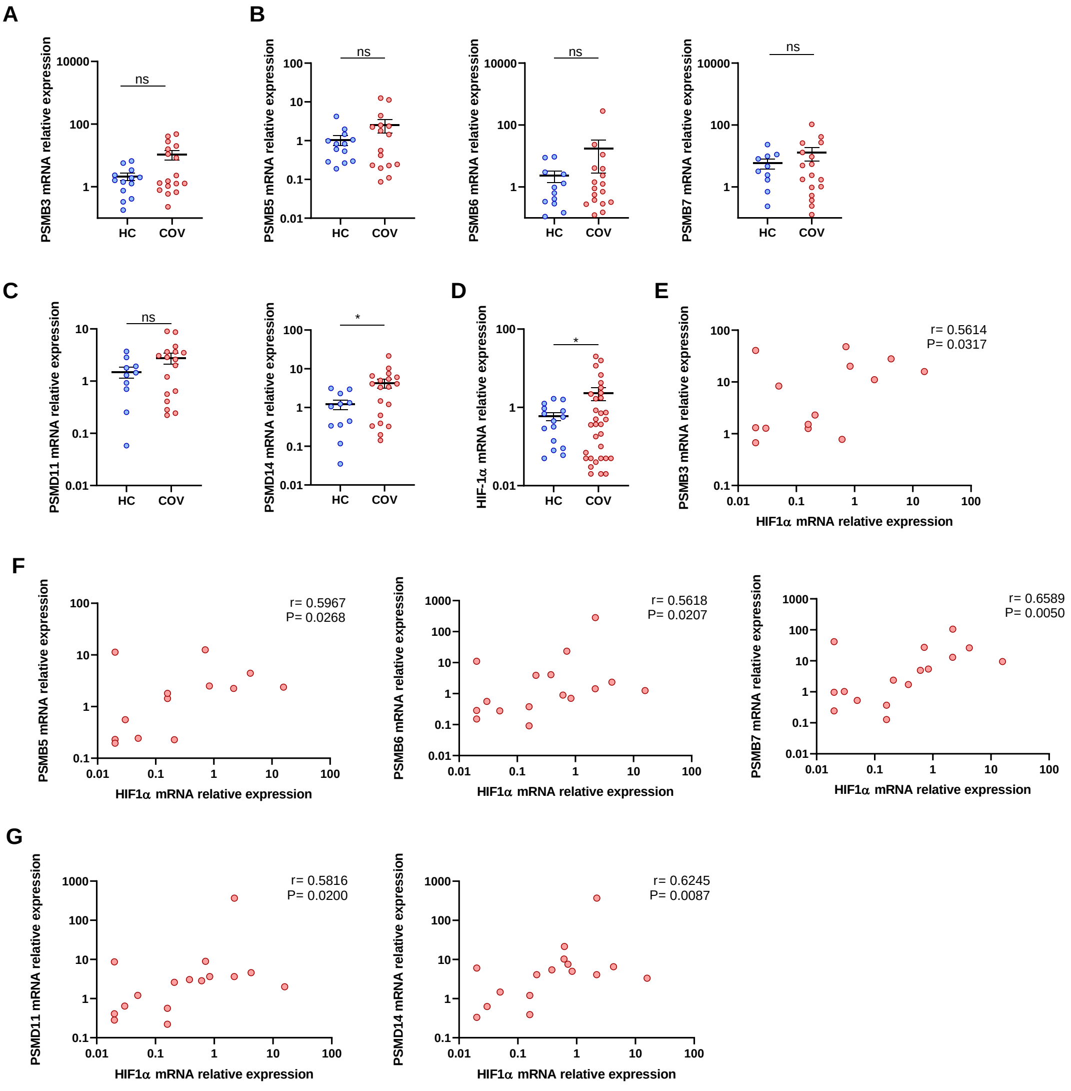


**Figure S1. Proteasome and HIF-1α upregulation in COVID-19 patients. (A-C)** mRNA expression in PBMCs from healthy controls (HC) compared to COVID-19 patients (COV) of **(A)** 20s proteasome beta ring non-catalytic subunits: PSMB3 (HC n=13; COV n=17); **(B)** 20S proteasome beta ring catalytic subunits: PSMB5 (HC n=13; COV n=16), PSMB6 (HC n=12; COV n=19), PSMB7 (HC n=11; COV n=19); **(C)** 19S proteasome subunits: PSMD11 (HC n=10; COV n=17), PSMD14 (HC n=11; COV n=19); and **(D)** HIF-1α (HC n=15; COV n=32). Mean differences were analysed using unpaired Student's t-test analysis with Welch's correction. Error bars: standard error of the mean. *:p<0.05. **(E-G)** Correlation of hypoxia-inducible factor 1-alpha (HIF-1α) mRNA expression and mRNA expression of **(E)** PSMB3 (n=15), **(F)** PSMB5 (n=14), PSMB6 (n=17), PSMB7 (n=17); and **(G)** PSMD11(n=16), PSMD14(n=17). Correlations were analysed using Spearman's analysis. P-value and Spearman's correlation coeficient (ρ) are shown.

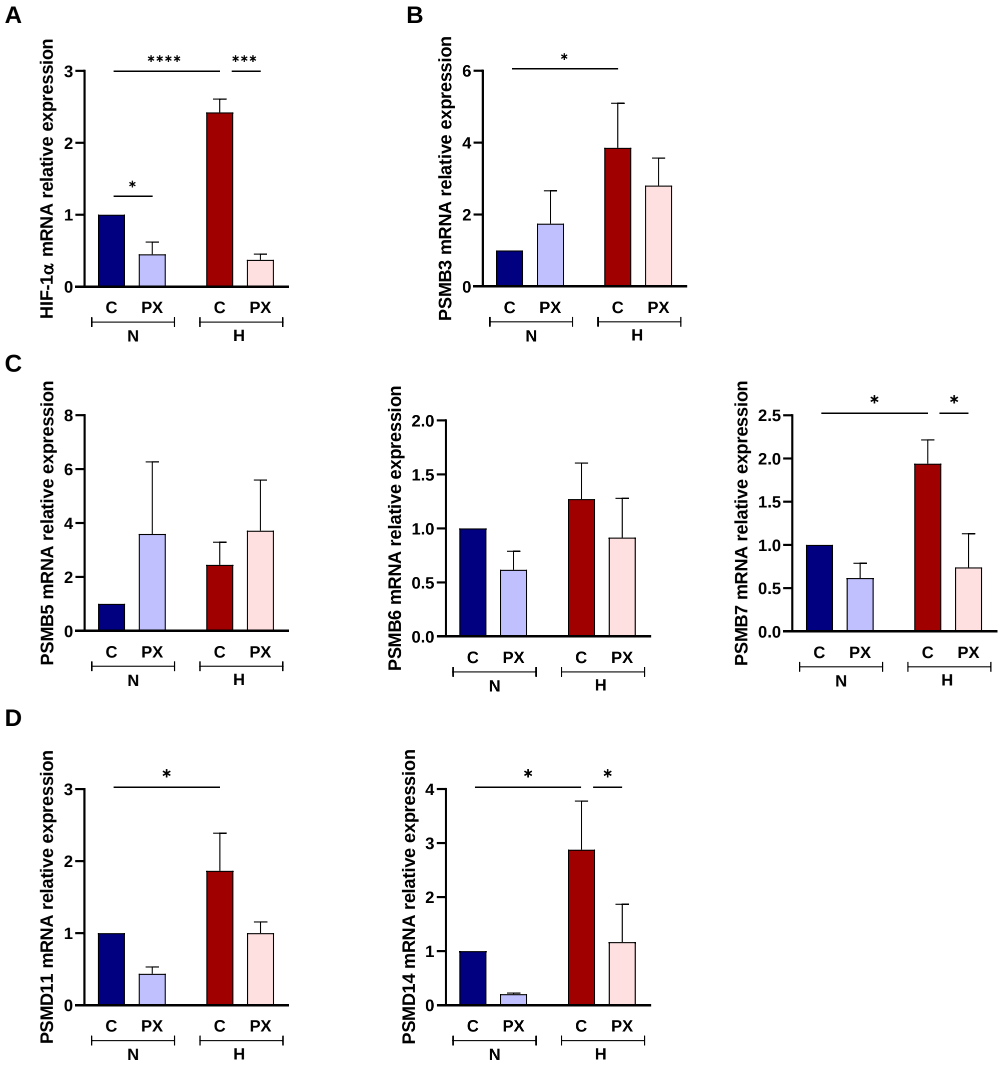


**Figure S2. *In vitro* model of the effect of hypoxia on proteasome subunits expression.** mRNA expression from healthy volunteers PBMCs (n=5) cultured in normoxia (N) or hypoxia (H) and treated with PX478 (PX) or untreated (C) of **(A)** HIF-1α; **(B)** 20S proteasome beta ring non-catalytic subunits: PSMB3; **(C)** 20S proteasome beta ring catalytic subunits: PSMB5, PSMB6, PSMB7; and **(D)** 19S proteasome subunits: PSMD11, PSMD14. Mean differences were analysed using mixed-effects analysis with Bonferroni's multiple comparison test. Error bars: standard error of the mean. *:p<0.05, ***:p<0.001, ****:P<0.0001.

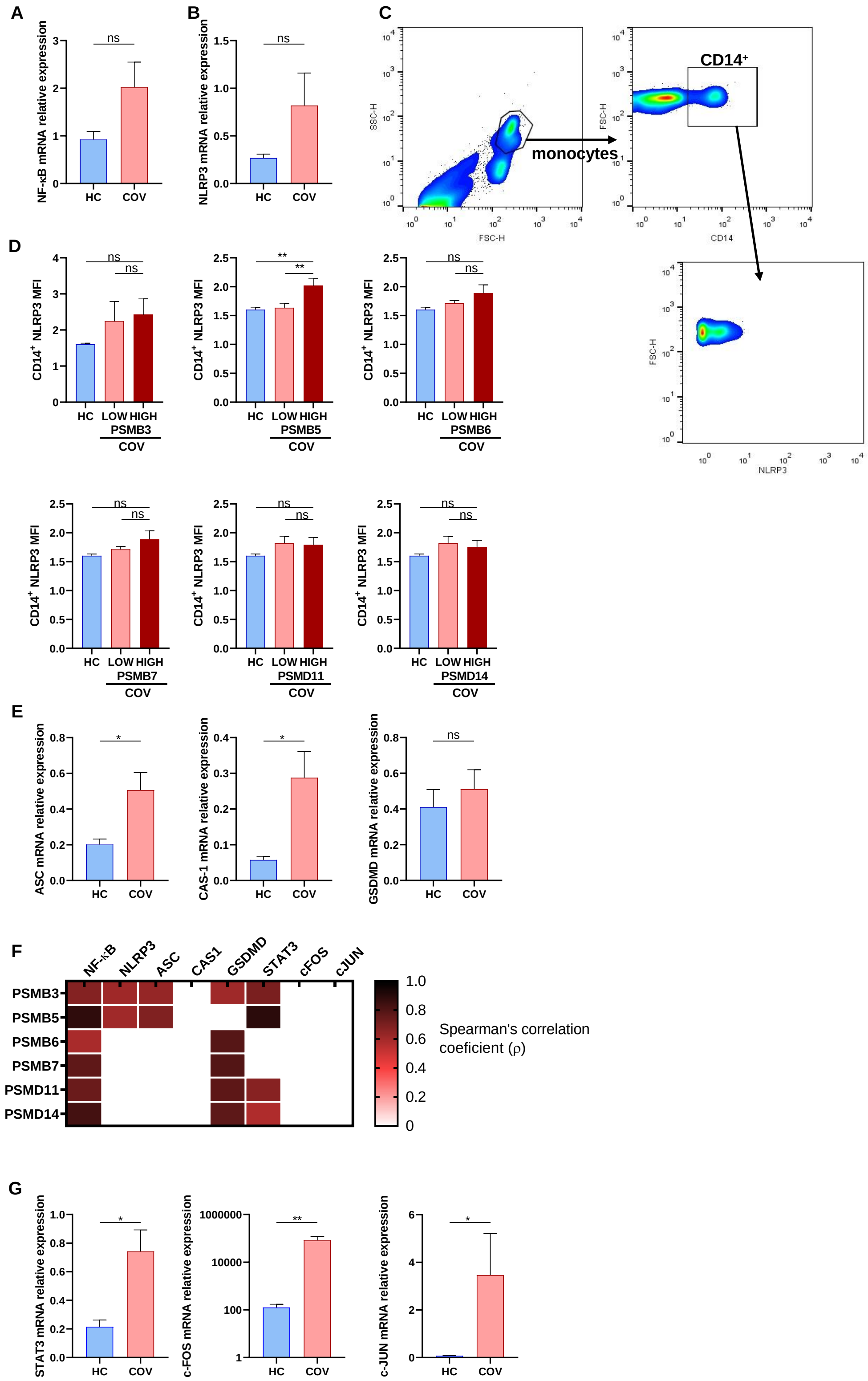


**Figure S3. Elevated mRNA expression of genes related to inflammation or STAT3 pathway in COVID-19 patients. (A)** NF-κB mRNA expression in healthy control (HC, n=12) and COVID-19 patients (COV, n=40), **(B)** NLRP3 mRNA expression in HC (n=18) and COV (n=44). **(C)** Monocyte gating strategy: representative plots demonstrating the used gating strategy to compare the NLRP3 expression. **(D)** NLRP3 expression in monocytes analysed by flow cytometry in monocytes from healthy controls (HC),

and COVID-19 patients (COV) presenting LOW or HIGH expression of proteasome subunits. Mean differences were analysed using one-way ANOVA analysis with Tuckey's multiple comparisons test. Error bars: standard error of the mean. *: $p<0.05$, **: $p<0.01$. **(E)** Comparison of NLRP3 inflammasome components mRNA expression in healthy control (HC) and COVID-19 patients (COV): ASC (HC, n=20; COV, n=44), CAS-1 (HC, n=20; COV, n=44) and GSDMD (HC, n=12; COV, n=43). **(F)** Heatmap represents Spearman's correlation coefficient (ρ) between proteasome subunits mRNA expression and genes related to inflammation or STAT-3 pathway mRNA expression. White gaps represent non-significant correlations, on the contrary the rest of correlations are significant ($p<0.05$). (**G)** STAT3 mRNA expression from PBMCs of healthy control (HC, n=14) and COVID-19 patients (COV, n=39), c-FOS mRNA expression in HC (n=14) and COV (n=39), c-JUN mRNA expression in HC (n=14) and COV (n=37). Mean differences were analysed using unpaired Student t-test analysis with Welch correction. Error bars: SEM. *: $p<0.05$, **: $p<0.01$.

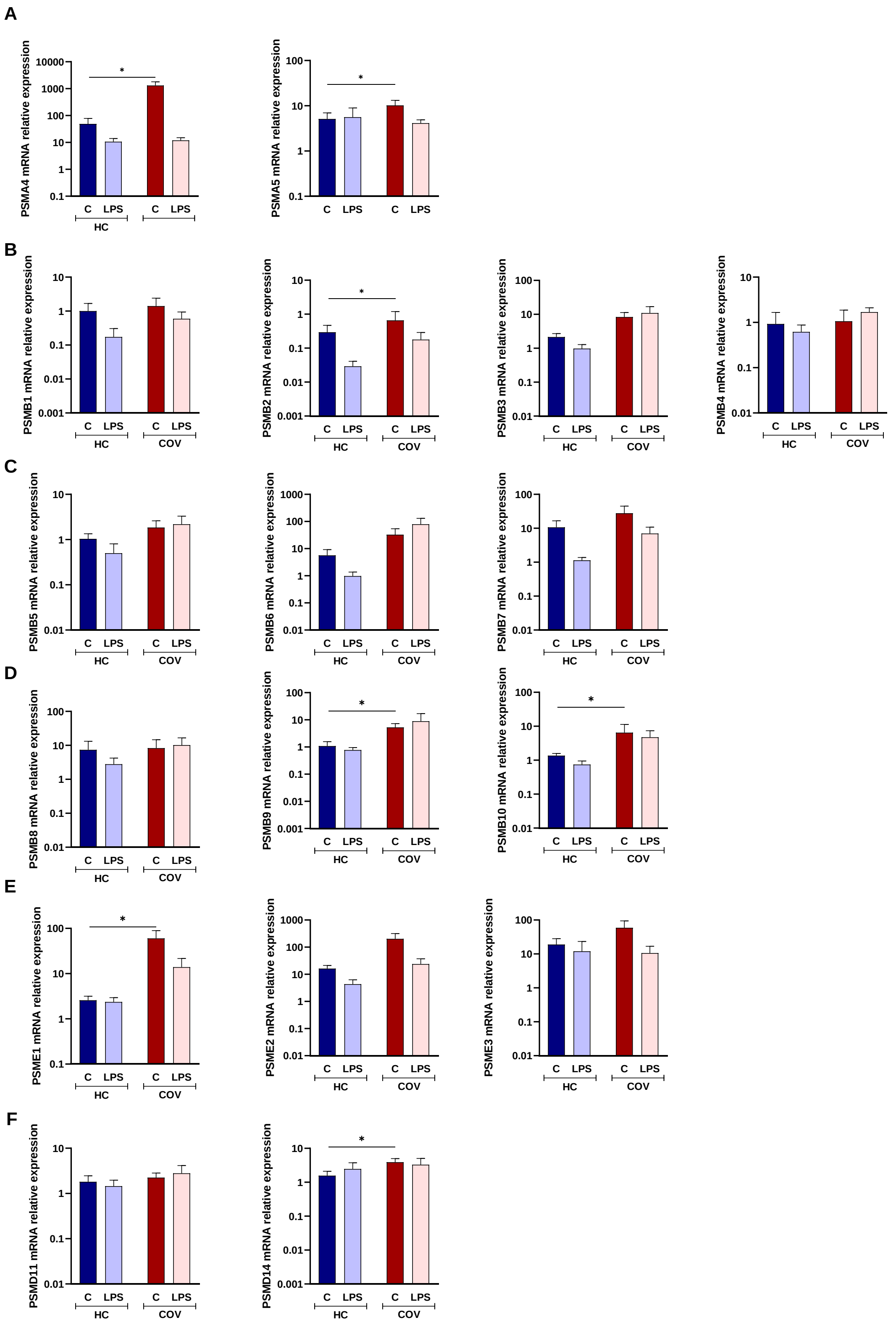


**Figure S4.** ***In vitro*** **model of the effect of inflammatory response is proteasome subunits expression**. Proteasome subunits mRNA expression in PBMCs from healthy controls (HC) (n=12) or COVID-19 patients (COV) (n=24) cultured for 16h under control conditions (C) or treated with 10ng/ml of LPS (LPS). **(A)** 20S proteasome alpha ring subunits: PSMA4, PSMA5; **(B)** 20S proteasome beta ring non-catalytic subunits: PSMB1, PSMB2, PSMB3, PSMB4; **(C)** 20S proteasome beta ring catalytic subunits: PSMB5, PSMB6, PSMB7; **(D)** 20S immunoproteasome beta ring catalytic subunits: PSMB8, PSMB9, PSMB10;

**(E)** 11S proteasome subunits: PSME1, PSME2, PSME3; and **(F)** 19S proteasome subunits: PSMD11, PSMD14. Mean differences were analysed using mixed-effects analysis with Bonferroni's multiple comparison test. Error bars: standard error of the mean. *: $p<0.05$.
